# Toll-like receptor 4 contributes to the inhibitory effect of morphine on colonic motility *in vitro* and *in vivo*

**DOI:** 10.1038/srep09499

**Published:** 2015-03-26

**Authors:** Aitak Farzi, Juraj Halicka, Raphaela Mayerhofer, Esther E. Fröhlich, Eva Tatzl, Peter Holzer

**Affiliations:** 1 Research Unit of Translational Neurogastroenterology, Institute of Experimental and Clinical Pharmacology, Medical University of Graz, Universitätsplatz 4, 8010 Graz, Austria; 2 Department of Pathophysiology, Jessenius Faculty of Medicine, Comenius University in Bratislava, Martin, Slovakia; 3 Department of Anaesthesiology and Intensive Care Medicine, Medical University of Graz, Auenbruggerplatz 29, 8036 Graz, Austria

## Abstract

Opioids rank among the most potent analgesic drugs but gastrointestinal side effects, especially constipation, limit their therapeutic utility. The adverse effects of opioids have been attributed to stimulation of opioid receptors, but emerging evidence suggests that opioids interact with the innate immune receptor Toll-like receptor 4 (TLR4) and its signalling pathway. As TLR4 signalling affects gastrointestinal motility, we examined the involvement of TLR4 in morphine-induced depression of peristaltic motility in the guinea-pig intestine *in vitro* and male C57BL/6N mice *in vivo*. While the TLR4 antagonist TAK-242 (0.1 μM and 1 μM) did not alter the morphine-induced inhibition of peristalsis in the isolated guinea-pig small intestine, the morphine-induced decrease in pellet propulsion velocity in colonic segments was attenuated by TAK-242 (0.1 μM). The ability of TAK-242 (4 mg/kg) to mitigate the morphine-induced suppression of colonic motility was replicated in mice *in vivo* by measuring the expulsion time of beads inserted in the distal colon. The inhibition of upper gastrointestinal transit of mice by morphine was not affected by pre-treatment with TAK-242 (4 mg/kg) *in vivo*. This is the first report that morphine-induced inhibition of colonic peristalsis is alleviated by TLR4 antagonism. We therefore conclude that TLR4 may contribute to opioid-induced constipation.

Opioids such as morphine rank among the most potent analgesic drugs, but their therapeutic value is limited due to side effects, especially in the gastrointestinal (GI) tract^1^. While the analgesic effects of opioids are primarily mediated via action within the central nervous system, their constipating effect is mainly attributed to activation of receptors within the GI tract^1^,^2^. Opioid-induced constipation (OIC) is the most common side effect and has been reported to occur in up to 95% of patients^3^, while a meta-analysis revealed that 41% of patients receiving opioids for non-cancer pain suffer from OIC^4^. Furthermore, the GI side effects are perceived as the most bothersome and can lead to discontinuation of opioid therapy^5^.

Opioids have hitherto been thought to exert their actions exclusively through stimulation of G-protein-coupled receptors (GPCRs) which are categorized into three major groups (μ-, δ-, and κ-opioid receptors)^6^. Opioid receptors, primarily of the μ type, are expressed in the myenteric plexus of the enteric nervous system (ENS), which is of paramount importance for controlling gut motility, and so far OIC has been related mainly to stimulation of myenteric opioid receptors^1^. The mechanisms whereby opioids inhibit intestinal motility comprise suppression of enteric nerve activity and release of neurotransmitters^1^.

Emerging evidence, however, indicates that select opioids not only activate genuine opioid receptors, but also the innate immune receptor Toll-like receptor 4 (TLR4), which plays a pivotal role in innate immunity by promoting the production of proinflammatory cytokines^7^,^8^. Unlike the stereoselective stimulation of opioid receptors, the interaction of opioid receptor agonists and antagonists with TLR4 takes place in a non-stereoselective manner^9^. This interaction has been demonstrated to mediate neuroinflammatory responses to opioids, to oppose the analgesic action of opioids and to contribute to opioid tolerance^10^,^11^. *In silico* docking studies uncovered that the soluble TLR accessory protein MD-2 is the preferred docking site of opioids^8^.

In addition to the interaction of opioids with TLR4 in the nervous system, the μ-opioid receptor agonist morphine has been demonstrated to cause intestinal epithelial barrier dysfunction in a TLR4- and μ-opioid receptor (MOR) dependent manner^12^. In this context, an intracellular cross talk between MOR and TLR4 has been proposed, as morphine-induced bacterial translocation was abolished and attenuated in MOR- and TLR4 knockout (KO) mice, respectively^12^.

In addition to the effects on epithelial barrier function, TLR4 signalling seems to be involved in disturbances of gut motility^13^. Thus, neurons of the myenteric and submucosal plexus express TLR4^14^,^15^, and sublethal concentrations of lipopolysaccharide (LPS), a bacterial cell membrane component activating TLR4, are frequently used to model sepsis-induced ileus^16^. It has not yet been investigated, however, whether TLR4 plays a role in the inhibitory effect of opioids on propulsive motility in the GI tract. Therefore, this study set out to examine the effect of TLR4 antagonism on morphine-induced depression of peristaltic motility in the guinea-pig intestine *in vitro* and mouse *in vivo*.

## Results

### TLR4 antagonism with TAK-242 blunts the morphine-induced retardation of pellet propulsion velocity in the isolated guinea-pig colon

**Figure 1:**
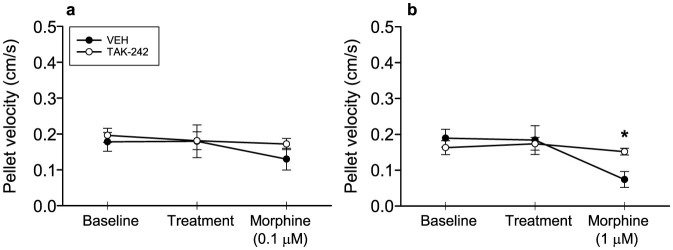
The TLR4 antagonist TAK-242 blunts morphine-induced retardation of pellet propulsion velocity in the isolated guinea-pig colon. Colon segments were exposed to vehicle (VEH) or the TLR4 antagonist TAK-242 (0.1 μM) for 10 min and pellet propulsion velocity was assessed. Subsequently morphine 0.1 μM (a) or 1 μM (b) was added to the bath and pellet propulsion velocity assessed again after 10 min. The values are means ± SEM, n = 7–8; *p < 0.05 versus VEH.

Morphine (0.1–1 μM) concentration-dependently decreased pellet propulsion velocity (Fig. 1a, b). The effect of 1 μM morphine (F_(2, 26)_ = 10.732, p < 0.001) was significantly mitigated by TAK-242 (0.1 μM) as there was a significant interaction between TAK-242 and 1 μM morphine (F_(2, 26)_ = 5.056, p = 0.014) (Fig. 1b). The lower dose of morphine (0.1 μM) likewise retarded pellet propulsion velocity (F_(2, 22)_ = 4.229, p = 0.028) but without an interaction with TAK-242 (Fig. 1a). Pre-treatment with TAK-242 (0.1 μM) *per se* had no effect on pellet propulsion velocity.

### TLR4 antagonism with TAK-242 does not alter morphine-induced inhibition of peristalsis in the guinea-pig small intestine

**Figure 2:**
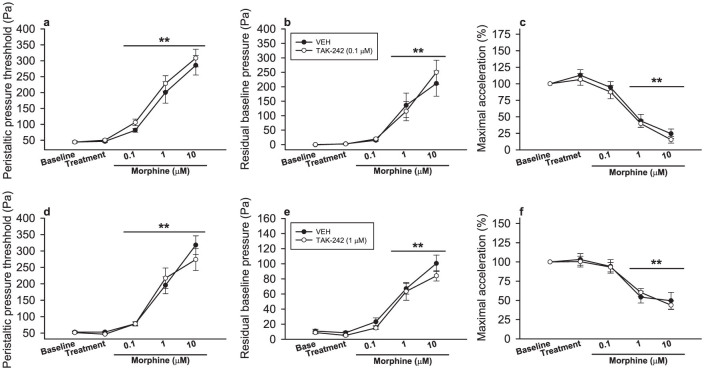
The TLR4 antagonist TAK-242 does not modify morphine-induced inhibition of peristalsis in the isolated guinea-pig small intestine. Small intestinal segments were exposed to vehicle (VEH) or the TLR4 antagonist TAK-242 (a–c: 0.1 μM, d–f: 1 μM) for 15 min, and peristaltic pressure threshold (a, d), residual baseline pressure (b, e), and maximal acceleration of the peristaltic waves (baseline set as 100%) (c, f) were assessed. Subsequently, increasing doses of morphine were added to the organ bath (yielding concentrations of 0.1–10 μM) in a cumulative manner at 15 min intervals, and the parameters of peristalsis were measured after each dose increment. The values are means ± SEM, n = 13–14 (a–c), n = 10–11 (d–f); **p < 0.001 versus baseline.

Morphine (0.1–10 μM) concentration-dependently enhanced PPT (F_(1.6, 39.1)_ = 104.100, p < 0.001) and the residual baseline pressure (F_(1.7, 40.1)_ = 37.559, p < 0.001) and reduced the maximal acceleration (F_(2.2, 50.1)_ = 76.589, p < 0.001) of the peristaltic waves (Fig. 2a–c). Pre-treatment with TAK-242 (0.1 μM) failed to modify *per se* any of the peristalsis parameters and did not alter the inhibitory effect of morphine on peristalsis (Fig. 2a–c) as there was no interaction between TAK-242 and morphine in any of the peristalsis parameters investigated.

Likewise, experiments with a higher dose of TAK-242 (1 μM) did not reveal any effects of TAK-242 on the peristalsis parameters or an interaction between TAK-242 and morphine (Fig. 2d–f). As in the experiment described above, morphine (0.1–10 μM) concentration-dependently enhanced PPT (F_(1.8, 33.3)_ = 78.161, p < 0.001) and the residual baseline pressure (F_(2.2, 24.0)_ = 68.991, p < 0.001) and reduced the maximal acceleration (F_(4, 44)_ = 28.418, p < 0.001) of the peristaltic waves (Fig. 2d–f).

### TLR4 antagonism with TAK-242 blunts the morphine-induced retardation of murine colorectal propulsion *in vivo*

**Figure 3:**
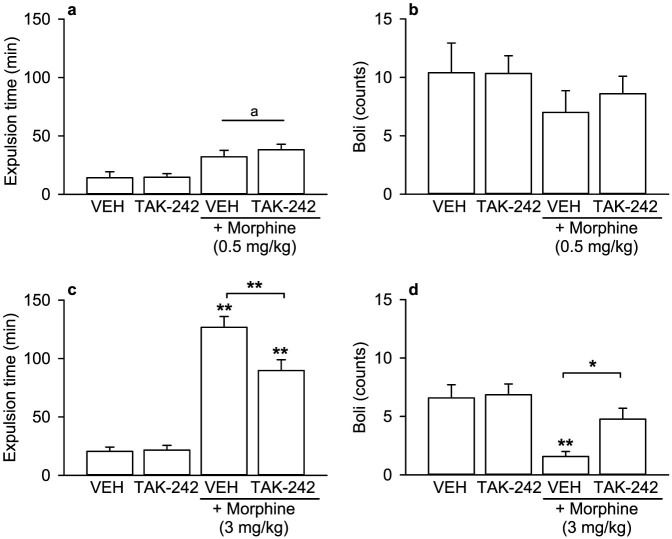
The TLR4 antagonist TAK-242 blunts morphine-induced inhibition of murine colonic propulsion *in vivo*. Mice were injected i.p. with vehicle (VEH) or the TLR4 antagonist TAK-242 (4 mg/kg), and 30 min later saline or morphine (0.5 and 3 mg/kg as indicated) was injected s.c. A ceramic bead was inserted into the colon 50 min after injection of morphine and the latency to expulsion recorded. The number of faecal pellets was assessed 3 h after morphine injection (b, d). The values are means + SEM; n = 5–6 (a, b), n = 13–14 (c, d). Post-hoc analysis of significant interactions in 2-way ANOVA: *p < 0.05, **p < 0.001 versus VEH or TAK-242 or as indicated by the bracket. Main factor effect without interactions: ^a^p < 0.001, morphine versus no morphine.

Subcutaneous injection of 0.5 or 3 mg/kg morphine led to a dose-dependent increase of bead expulsion time (Fig. 3a, c) which was accompanied by a decrease of faecal pellet excretion (Fig. 3b, d). Pre-treatment with the TLR4 antagonist TAK-242 (4 mg/kg) blunted the effects of 3 mg/kg, but not 0.5 mg/kg morphine on distal colonic motility (Fig. 3a, c). On its own, TAK-242 failed to modify any of the parameters measured (Fig. 3a–d).

Two-way ANOVA of the effects of 0.5 mg/kg morphine revealed a significant main factor effect of morphine treatment on bead expulsion time (F_(1, 17)_ = 19.007, p < 0.001) while there was no significant interaction between TAK-242 and morphine (Fig. 3a). Likewise, TAK-242 and 0.5 mg/kg morphine had no significant influence on faecal pellet expulsion (Fig. 3b).

Statistical analysis revealed a significant interaction between TAK-242 and 3 mg/kg morphine in the changes of bead expulsion time (F_(1, 49)_ = 6.951, p = 0.011) (Fig. 3c). Post-hoc analysis disclosed that, while bead expulsion time was increased by morphine in the absence and presence of TAK-242, the morphine-induced inhibition of distal colonic motility was significantly alleviated by TAK-242 (p < 0.001) (Fig. 3c). The interaction between TAK-242 and 3 mg/kg morphine in the change of faecal pellet counts approached significance (F_(1, 50)_ = 3.269, p = 0.077) (Fig. 3d). Post-hoc analysis revealed that faecal pellet production was solely suppressed by morphine alone, while pre-treatment with TAK-242 was able to attenuate the inhibition of faecal pellet production by morphine (Fig. 3d).

### TLR4 antagonism with TAK-242 does not alter morphine-induced inhibition of murine upper GI transit *in vivo*

**Figure 4:**
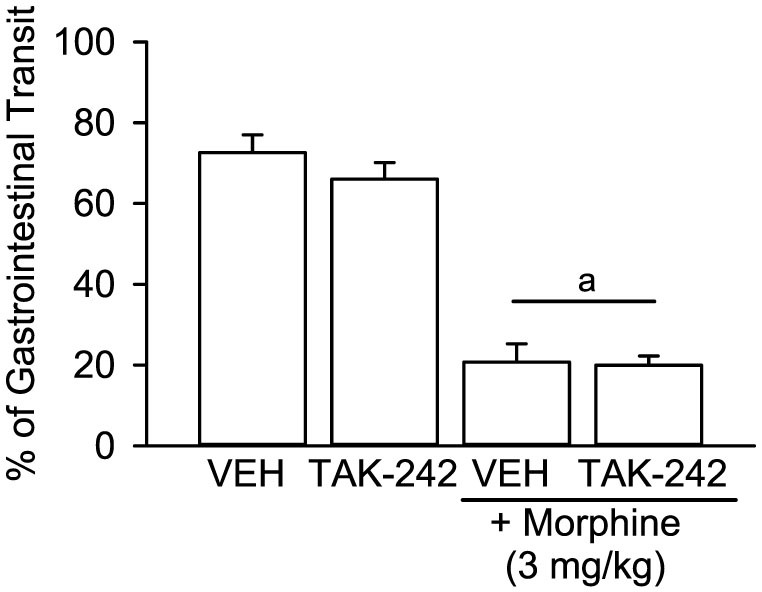
The TLR4 antagonist TAK-242 does not modify morphine-induced inhibition of murine upper GI transit *in vivo*. Mice were injected i.p. with vehicle (VEH) or the TLR4 antagonist TAK-242 (4 mg/kg), and 30 min later saline or morphine (3 mg/kg) was injected s.c. Twenty min after injection of morphine, 5% Evans blue was gavaged. Upper GI transit is expressed as percentage of the distance travelled by the Evans blue bolus relative to the total length of the small intestine, measured 30 min after Evans blue administration. The values are means + SEM; n = 8–9. Main factor effect without interactions: ^a^p < 0.001, morphine versus no morphine.

In contrast to the attenuation of morphine-induced retardation of colonic propulsion, TAK-242 (4 mg/kg) had no effect on the inhibition of upper GI transit caused by morphine (3 mg/kg) (Fig. 4).

While subcutaneous injection of 3 mg/kg morphine led to a decrease of upper GI transit, the TLR4 antagonist TAK-242 failed to modify upper GI transit in vehicle- and morphine-treated mice.

Two-way ANOVA revealed a significant main factor effect of morphine treatment on upper GI transit (F_(1, 27)_ = 147.100, p < 0.001) while there was no significant interaction between TAK-242 and morphine (Fig. 4).

## Discussion

To the best of our knowledge this is the first study to reveal an involvement of TLR4 signalling in morphine-induced inhibition of intestinal motility. Two features of this relationship are particularly worth noting. First, the participation of TLR4 in the inhibitory motor effect of morphine is region-specific, given that the doses of the TLR4 blocker TAK-242 used here attenuated opioid-induced inhibition of propulsive motility in the colon but not in the small intestine. Second, the contribution of TLR4 to opioid-induced motor inhibition depends on the dose of morphine, given that the motor responses to higher morphine doses are more strongly inhibited by TAK-242 than those to lower morphine doses. Our findings add to the growing body of literature reporting an interaction of opioids with TLR4 in several organ systems including the GI tract, where morphine-induced epithelial barrier dysfunction and bacterial translocation seem to be mediated by TLR4 signalling^12^. In a similar perspective, the present findings provide a hint that OIC may also in part depend on TLR4 signalling.

The TLR4 antagonist used in this study, TAK-242 (resatorvid), is a small synthetic molecule inhibiting intracellular TLR4 signalling. Specifically, TAK-242 binds to the amino acid Cys747 in the intracellular domain of TLR4^17^,^18^,^19^ and disrupts the association of TLR4 with adaptor molecules, leading to the inhibition of signal transduction^20^. In line with its ability to disrupt TLR4 signalling, TAK-242 has been demonstrated to inhibit LPS-induced cytokine release and to attenuate LPS-induced relaxation of the longitudinal muscle of the guinea-pig colon^21^.

Differences in the degree to which TLR4 antagonism blunted morphine-induced inhibition of intestinal motility were seen between the *in vitro* and *in vivo* setting. Thus, while the inhibitory effect of morphine on pellet propulsion velocity in the isolated guinea-pig colon was prevented by TAK-242, the inhibitory effect of morphine in the mouse colon was only partially reversed by a dose of TAK-242 found effective in other studies^47^. These results raise the question to which extent opioid receptors and TLR4 contribute to the inhibitory effect of morphine on colonic motility. Spontaneous faecal pellet excretion by segments of the guinea-pig colon has been reported to be retarded by morphine and reversed by the opioid antagonist naloxone^22^. Indeed, peripherally acting MOR antagonists such as naloxone are used to attenuate OIC^1^, but this compound does not allow to differentiate between opioid receptor- and TLR4-mediated effects because naloxone can antagonize TLR4 signalling as well^9^. Studies in MOR-KO mice, however, confirm that the inhibition of GI transit by morphine depends on MOR expression^23^. Therefore the *in vivo* results might give a more physiological picture of a partial involvement of TLR4 in OIC. Interestingly, morphine-induced inhibition of colorectal motility has been demonstrated to involve spinal and supraspinal sites of action^24^, offering an explanation for the differences between the *in vitro* and *in vivo* results. However, species differences in the contribution of TLR4 and MOR to opioid-induced intestinal motor stasis and differences between the *in vitro* and *in vivo* setting with regard to the dose of TAK-242 and morphine reaching the intestine might also account for the differences in the extent of TLR4 antagonism to blunt morphine-induced inhibition of colonic motility.

Two scenarios might underlie the attenuation of morphine-induced inhibition of colonic motility by TLR4 antagonism. On the one hand, morphine could bind directly to TLR4 and activate TLR4 signalling pathways as shown in the brain^11^. On the other hand, TLR4 and MOR signalling could interact with each other without binding of morphine to TLR4 as shown for intestinal epithelial barrier dysfunction in response to morphine^12^.

Already in the early era of opioid research non-stereoselective binding sites of opioids were discovered and non-classical actions of opioids described, but these aspects of opioid pharmacology received little attention until recently^10^,^25^. Only during the past decade it emerged that, apart from actions at genuine opioid receptors, opioids are also able to impact TLR4 which plays a pivotal role in innate immunity^7^,^8^. The pathophysiological relevance of this novel aspect of opioid action is just beginning to unfold, but seems to have implications in many of the adverse effects of opioids such as opioid resistance and tolerance, opioid-induced hyperalgesia and inflammatory reactions^10^,^11^,^26^.

There are several mechanisms whereby TLR4 signalling can affect intestinal motility and its control systems. For instance, the ENS can indirectly be modulated by immune cells producing inflammatory mediators such as proinflammatory cytokines, prostaglandins and nitric oxide, which affect neuronal activity^27^. Especially the MyD88-dependent signalling pathway downstream of TLR4 has been demonstrated to be involved in LPS-induced ileus^28^. In addition, direct TLR4 signalling within non-hematopoietic cells has been shown to play a substantial role in LPS-induced ileus^13^. However, the implications of TLR4 in ENS function are complex, given that TLR4 knockout is associated with a delay of intestinal transit and a decrease of nitrergic neurons, which may reflect a role of TLR4 in promoting neuronal survival in the ENS^29^. Furthermore, afferent neurons and the gut-brain axis have been involved in LPS-induced ileus^30^. As TAK-242 was able to blunt morphine-induced inhibition of colonic motility also in isolated intestinal segments, the involvement of extrinsic afferent neurons seems rather unlikely. Therefore, it could be envisaged that, in addition to binding to MOR, morphine also activates TLR4 signalling and thereby blunts colonic motility. However, while morphine seems to synergize with TLR4 to increase bacterial translocation, it has been reported to exert anti-inflammatory actions within the gut, attenuating cytokine levels in the context of intestinal inflammation^31^,^32^.

In explaining the region-specific participation of TLR4 in the morphine-induced inhibition of intestinal peristalsis it is important to consider that TLR4 signalling varies along the GI tract. For instance, the TLR-mediated dysfunction of the epithelial barrier which morphine can elicit in the murine gut is observed in the small intestine, but not colon^12^. TLR4-mediated motor effects of LPS likewise vary along the alimentary canal. Thus, while the peristaltic motor effects of LPS in the colon are mostly inhibitory, TLR4 signalling in the small intestine can either stimulate or delay propulsive motility^16^.

These findings are in keeping with the ability of TAK-242 to alleviate the antiperistaltic effect of morphine especially in the colon as seen in the present study. This regional variation in the effect of TAK-242 may be explained by regional differences in the expression of TLR4 which in the colon is higher than in the small intestine^15^,^33^. In addition, the effect of morphine on intestinal motility is also subject to regional differences, because the motor response of the murine distal colon to morphine is substantially stronger than that of the murine small intestine^34^. It remains to be investigated to which extent the transduction systems operated by opioids vary in the different regions of the GI tract.

The contribution of the innate immune receptor TLR4 to the antiperistaltic action of morphine in the colon, but not small intestine, is interesting in another perspective. The colon is inhabited by the largest microbiota community in the body, and this community is known to closely interact with the intestinal immune system^35^. Signal transduction mechanisms involving TLRs may therefore play particular roles in this part of the GI tract. Opioid peptides are not only produced by enteric neurons and GI epithelial cells, but also by GI immune cells and, as indirect evidence suggests, by members of the gut microbiota^1^,^36^,^37^. Given the expression of TLRs by immune and non-immune cells, signalling via TLRs in the colon may be a mechanism that is called into operation by excess concentrations of messenger molecules such as opioid receptor agonists. The pathophysiological relevance of this scenario awaits to be elucidated.

As alluded to before, it was a consistent observation in this study that TAK-242 mitigated the antiperistaltic effect of higher, but not lower, doses of morphine. Conceptually, this finding could indicate that TLR4 signalling is operated by morphine only at concentrations that are higher than those required to stimulate genuine opioid receptors. Another explanation for this dose-dependent implication of TLR4 in the morphine-induced motor inhibition comes from a molecular analysis of the interaction of morphine with TLR4 on NF-κB reporter cells^38^. In this study, select concentrations of morphine were found to activate TLR4, yielding a bell-shaped dose-response curve. On the basis of these results, morphine was described as a weak partial agonist at TLR4^38^.

In contrast to binding and activating TLR4, morphine has also been demonstrated to affect TLR4 expression^39^. Thus, an increased expression of TLR4 in the small intestine by stimulation of opioid receptors has been proposed to be implicated in morphine-induced epithelial barrier dysfunction^12^. Interestingly, TLR4 expression in the colon was not affected by morphine^12^. Therefore, changes in TLR4 expression are rather unlikely to occur in the present study, especially when considering that the effects were observed within a short time after treatment. However, TLR4 activation has also been demonstrated to impact on MOR expression and signalling^40^.

As a GPCR, MOR is regulated by G protein-coupled receptor kinases (GRKs). Typically, GRKs, in combination with β-arrestins, are able to desensitize GPCRs and thereby hamper their responsiveness to agonists. However, under certain conditions GRK6 and β-arrestin-2 also act as positive mediators of receptor signalling and stimulate mitogen-activated protein kinases and other signal transducers^41^. Intriguingly, both GRK6 KO and b-arrestin-2 KO mice have been found to expel more boli than wildtype (WT) mice in response to morphine^42^,^43^, as seen for TLR4 antagonism with TAK-242 in the current study. Likewise, the bead expulsion time in response to distinct doses of morphine in GRK6 KO and b-arrestin-2 KO mice was shortened relative to that in WT mice, while upper GI transit in response to morphine was not different between KO and WT mice^42^,^43^. TLR4 has also been demonstrated to interact with GRK6 and β-arrestin-2^44^, and the responses of the KO mice to the antiperistaltic effects of morphine resemble those of TLR4 antagonism in the current study. It is hence tempting to speculate that an interaction of TLR4 signalling with GRK6 and β-arrestin-2 is involved in the attenuation of the morphine-induced decrease of colonic motility by TLR4 antagonism. Specifically, TLR4 antagonism might block the interaction of TLR4 signalling pathways with positive mediators of MOR signalling such as GRK6 and β-arrestin-2 and in this way blunt MOR signalling in the colon. Obviously, this hypothesis requires further analysis.

In summary, the current study provides pharmacological evidence that TLR4 signalling participates in the effect of morphine to inhibit propulsive motility, particularly in the colon. This relationship was revealed by the TLR4 blocker TAK-242 which attenuated the antiperistaltic effect of morphine in the isolated guinea-pig colon, an effect that was confirmed in the murine colon *in vivo*. In a translational perspective, our findings suggest that TLR4 signalling contributes to OIC and that selective TLR4 blockers may have therapeutic potential as adjuncts to opioid therapy, alleviating adverse effects of opioids such as constipation.

## Methods

### Experimental animals

Experiments on the isolated intestine were performed with segments excised from adult male TRIK strain guinea-pigs weighing 350–450 g. The guinea-pigs were provided by the Department of Toxicology and Laboratory Animal Breeding, Institute of Experimental Pharmacology of the Slovak Academy of Sciences, Dobrá Voda, Slovakia.

The *in vivo* experiments were carried out with male C57BL/6N mice obtained from Charles River Laboratories (Sulzfeld, Germany) at the age of 13 weeks. The animals were kept in groups of 4 in the institutional animal house. Light conditions (lights on at 6:00 h, lights off at 18:00 h), temperature (set point 22°C) and relative air humidity (set point 50%) were tightly controlled. Standard laboratory chow and tap water were provided ad libitum.

### Ethics statement

The experimental procedures and number of animals used were approved by an ethical committee at the Federal Ministry of Science and Research of the Republic of Austria (BMWF-66.010/0026-WF/II/3b/2014) and conducted according to the Directive of the European Parliament and of the Council of 22 September 2010 (2010/63/EU). The experiments were designed in such a way that the number of animals used and their suffering was minimized.

### Experiments on the guinea-pig small intestine *in vitro*

#### Recording of peristalsis

The small intestine was isolated, flushed of luminal contents and placed in Tyrode solution kept at room temperature and oxygenated with a mixture of 95% O_2_ and 5% CO_2_^45^. The composition of the Tyrode solution was (mM): NaCl 136.9, KCl 2.7, CaCl_2_ 1.8, MgCl_2_ 1.0, NaHCO_3_ 11.9, NaH_2_PO_4_ 0.4 and glucose 5.6. The small intestine was divided into eight segments, each being approximately 10 cm long. Segments were assigned randomly to the pharmacological treatments under study as the baseline peristaltic parameters did not significantly differ between segments. Four intestinal segments were set up in parallel in organ baths containing 30 ml of Tyrode solution at 37°C. In order to elicit propulsive peristalsis, prewarmed Tyrode solution was infused into the lumen of the segments at a rate of 0.5 ml/min. The intraluminal pressure at the aboral end of the segments was measured with a pressure transducer whose signal was fed into a personal computer^45^.

The fluid passing through the gut lumen was directed into a vertical outlet tubing which ended 4 cm above the fluid level in the organ bath. The aborally moving wave of peristaltic contraction (peristaltic wave) resulted in a spike-like increase in the intraluminal pressure, which caused emptying of the segment if the maximal pressure of the peristaltic wave exceeded the level of 400 Pa as set by the position of the outlet tubing^45^.

#### Experimental protocol

The TLR4 antagonist TAK-242 (resatorvid; ethyl (6R)-6-[N-(2-chloro-4-fluorophenyl)sulfamoyl]cyclohex-1-ene-1-carboxylate; Calbiochem/Merck Millipore, Darmstadt, Germany) was dissolved in dimethylsulfoxide (DMSO) yielding a 10 mM stock solution. The stock solution was further diluted with Tyrode solution and added to the organ bath resulting in a final concentration of 0.1 μM or 1 μM TAK-242. The concentration of 0.1 μM TAK-242 was based on pilot studies, which showed that this concentration was effective in attenuating the inhibition of colon motility induced by morphine *in vitro*, and on findings reported by other groups that 0.1 μM TAK-242 inhibits LPS-induced TNF-α production in RAW264.7 cells^19^. As 0.1 μM TAK-242 did not affect small intestinal motility, a higher concentration of TAK-242 (1 μM) was also tested in a separate experiment.

DMSO at the same volume and concentration was used as vehicle (VEH) control. Morphine hydrochloride (Diosynth, Apeldoorn, Holland) was dissolved in Tyrode solution.

The preparations were allowed to equilibrate in the organ bath for a period of 30 min and the baseline peristaltic activity was recorded for a 15 min period. Drugs to be tested were added to the bath, i.e., to the serosal surface of the intestinal segments, at volumes not exceeding 1% of the bath volume^45^.

The intestinal segments were exposed to VEH or TAK-242 15 min before morphine (0.1–10 μM) was added to the bath in a cumulative manner at 15 min intervals.

#### Evaluation of peristalsis

The recordings of peristalsis were analysed with the software “Peristal 1.0” with regard to three different parameters: the peristaltic pressure threshold (PPT), the residual baseline pressure, and the maximal acceleration of the peristaltic waves^45^. PPT (Pa) is the intraluminal pressure at which a peristaltic wave is triggered. The residual baseline pressure (Pa) equals the minimal intraluminal pressure that is achieved after completion of each peristaltic wave^45^. A further indicator of peristaltic effectiveness is the maximal acceleration of the peristaltic waves (Pa/s^2^), which is determined not only by the speed with which the muscle contracts but also by the speed with which the contraction moves aborally to empty the segments.

The peristaltic parameters of 3–4 peristaltic waves at the peak effect of the compounds under study were averaged.

### Experiments on the guinea-pig colon *in vitro*

The colon of the guinea-pigs was isolated and placed in Tyrode solution. Faecal pellets were allowed to excrete spontaneously from the segments. The colon was then divided into six segments, each being approximately 5 cm long. Three colon segments were set up in parallel in organ baths containing 50 ml of Tyrode solution maintained at 37°C and oxygenated with a mixture of 95% O_2_ and 5% CO_2_. The segments were assigned randomly to the pharmacological treatments.

The preparations were allowed to equilibrate in the organ bath for a period of 10 min. Subsequently, a wooden pellet (outer diameter 3 mm, length 1 cm) was introduced into the colon segment at the oral end. The velocity of pellet propulsion (cm/s) was calculated from the time taken by a pellet to move 4 cm from the oral end of the segment^46^.

The velocity of pellet propulsion was measured up to 8 times in each segment, the consecutive runs of propulsion measurement being performed at 5–10 min intervals. Initially 3 runs were conducted in order to record the baseline pellet propulsion velocity. Subsequently the drugs to be tested were added to the bath. First, TAK-242 at a concentration of 0.1 μM or its VEH was added to the bath for 10 min and the pellet propulsion velocity assessed. Subsequently morphine (0.1 or 1 μM) was added to the bath for 10 min and pellet propulsion time measured twice at 5 min intervals. Each experiment was carried out with at least six segments from six different guinea-pigs.

### Experiments on the murine small intestine and colon *in vivo*

#### Injection regimen

Morphine hydrochloride was dissolved in pyrogen-free sterile saline (0.9% NaCl) and administered subcutaneously (s.c.) at a dose of 0.5 or 3 mg/kg and an injection volume of 20 μl/10 g body weight. Pyrogen-free sterile saline at the same volume was used as VEH control.

TAK-242 was dissolved in DMSO yielding a 10 mM stock solution. The stock solution was further diluted with sterile saline and injected intraperitoneally (i.p.) at a dose of 4 mg/kg and an injection volume of 100 μl/10 g body weight. The dose of 4 mg/kg TAK-242 was based on pilot studies and reports of other groups^47^. DMSO at the same volume and concentration was used as VEH control. TAK-242 or its VEH was injected 30 min prior to morphine.

#### Upper gastrointestinal transit

The mice were fasted for 18 h before the assessment of GI transit time but had free access to water^42^,^48^. Twenty min after morphine-injection 0.1 ml of a mixture of Evans blue (5%) and methyl cellulose (0.5%) in phosphate-buffered saline (Sigma–Aldrich, Vienna, Austria) was gavaged^49^. Thirty min after the administration, mice were sacrificed by cervical dislocation. The small intestine was dissected out from the pyloric sphincter to the ileocaecal junction. The total length of the small intestine and the distance travelled by the head of the Evans blue bolus were measured. Upper GI transit was expressed as a percentage of the distance travelled by the head of the Evans blue bolus relative to the total length of the small intestine^50^.

#### Distal colonic motility

The mice were fasted for 18 h before the assessment of intestinal transit time but had free access to water^42^,^48^. Fifty min after the injection of morphine a ceramic bead with a diameter of 1.4 mm (Peqlab, Erlangen, Germany) was inserted into the colon of the mice 2 cm proximal to the anal opening with the help of a plastic Pasteur pipette lubricated with vaseline (Rösch & Handel, Vienna, Austria)^49^. After insertion of the bead the mice were placed singly in cages devoid of bedding, and the latency until expulsion of the bead was assessed. In addition, the number of faecal pellets expelled was counted 3 h after morphine injection.

#### Statistics

Statistical evaluation of the results was made with SPSS 22 (SPSS Inc., Chicago, Illinois, USA). Normality was tested with the Shapiro–Wilk normality test. The data obtained from the isolated intestinal segments were analysed by repeated measures analysis of variance (ANOVA). In case of sphericity violations the Greenhouse-Geisser correction was applied. p-Values were adjusted for multiple comparisons with the Bonferroni correction. The data obtained from the *in vivo* experiments were analysed by two-way ANOVA, one factor being TAK-242 and the other factor being morphine. Post-ANOVA analysis of group differences was performed by Tukey's post hoc comparison. Probability values of p < 0.05 were regarded as statistically significant. All data are presented as means ± SEM, n referring to the number of intestinal segments or mice in each group.
